# Synthesis, crystal structure and Hirshfeld surface analysis of bis­[4-(2-amino­eth­yl)morpholine-κ^2^ *N*,*N*′]di­aqua­nickel(II) dichloride

**DOI:** 10.1107/S2056989023001470

**Published:** 2023-02-23

**Authors:** Balasubramanian Chidambaranathan, Settu Sivaraj, Pandy Vijayamathubalan, Shanmugam Selvakumar

**Affiliations:** a PG and Research Department of Physics, Government Arts College for Men, (Autonomous), Chennai 600 035, Tamil Nadu, India; Università di Parma, Italy

**Keywords:** crystal structure, metal-organic, morpholine, hydrogen bond, IR, Hirshfeld surface

## Abstract

The title coordination compound, [Ni(C_6_H_14_N_2_O)_2_(H_2_O)_2_]Cl_2_, was synthesized by mixing 4-(2-amino­eth­yl)morpholine and nickel chloride in double-distilled water. The asymmetric unit comprises one half of an Ni^II^ cation (located on an inversion centre), one 4-(2-amino­eth­yl)morpholine ligand, one coordinated water mol­ecule and one chloride ion outside the metal coordination sphere. The nickel ion is in a octa­hedral environment of the N_4_O_2_ type, coordinating four N atoms from two 4-(2-amino­eth­yl)morpholine ligands and two *trans*-located O atoms from two water mol­ecules. The morpholine ligand was found disordered over two positions with a site occupancy ratio of 0.708 (8):0.292 (8). The crystal structure is consolidated by N—H⋯Cl, N—H⋯O, C—H⋯Cl and C—H⋯O hydrogen bonds. Hirshfeld surface analysis confirms that van der Waals inter­actions are prevalent in the crystal packing of the synthesized complex.

## Abstract 2

The title coordination complex, [Ni(C_6_H_14_N_2_O)_2_(H_2_O)_2_]Cl_2_, crystallizes in the *P*2_1_/*n* space group. The metal ion displays a slightly distorted octa­hedral geometry. The crystal structure is consolidated by N—H⋯Cl, N—H⋯O, C—H⋯Cl, C—H⋯O and O—H⋯Cl hydrogen bonding.

## 1. Chemical context

Morpholine has been recognized as a convenient ligand for the design of supra­molecular structures (Cvrtila *et al.*, 2012 ▸) since it is a ditopic heterocyclic mol­ecule that can coordinate the metal ion *via* one hetero atom, leaving the other free for linking to other mol­ecules either by coordination to metal ions (Gálvez Ruiz *et al.*, 2008 ▸; Willett *et al.*, 2005 ▸; Lapadula *et al.*, 2010 ▸; Clegg *et al.*, 2010 ▸) or by hydrogen bonding (Lian *et al.*, 2008 ▸; Weinberger *et al.*, 1998 ▸; Ivanov *et al.*, 2001 ▸). The coordination of a morpholine mol­ecule to a metal ion activates the morpholine mol­ecule as a hydrogen-bond donor by increasing the partial positive charge of the morpholine amine hydrogen. Coordination of two morpholine mol­ecules through nitro­gen lone pairs on a single metal centre can lead to a good bonding site for negatively charged small species. A prerequisite for this is that the morpholine ligands are bonded to the central ion in a *cis* configuration, which may be achieved by the use of chelating co-ligands. These will induce the binding of morpholine in a convenient configuration, so that its N—H groups form a pincer, which can bind guest mol­ecules in the second coordination sphere. Intriguingly, reports of morpholine-based metal–organic receptors are scarce (White *et al.*, 1999 ▸). Transition-metal ions can be an important source of magnetic moments, and when connected through proper bridging ligands, superexchange inter­actions can take place (Konar *et al.*, 2005 ▸). Parallel to the development of organic electro-optical (EO) and non-linear optical (NLO) materials [Lamshöft *et al.*, (2011 ▸)], a subject of great inter­est comprises metal–organic chromophores. 
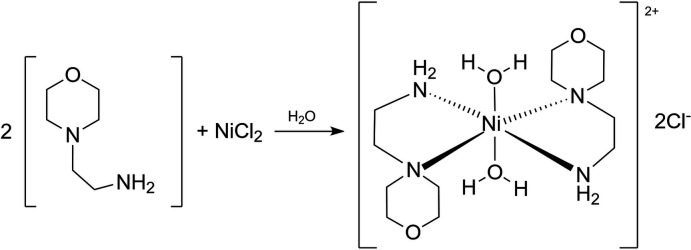


Metal–organic frameworks (MOFs) are crystalline hybrid materials with networks constructed from the self-assembly of metal ions with, at least, one organic linker. As a result of their availability from commercial sources and/or easy synthetic methodologies, organic ligands based on carboxyl­ate or nitro­gen compounds have been used extensively, mainly with transition-metal ions, to isolate new and improved MOF architectures, to be studied for a large range of practical applications (Horcajada *et al.*, 2012 ▸; Kreno *et al.*, 2012 ▸; He *et al.*, 2012 ▸; Chughtai *et al.*, 2015 ▸). Morpholine can be used as a ligand in metal complexes and it can also be a component of protective coatings on fresh fruits and used as an emulsifier in the preparation of pharmaceuticals and cosmetic products (Kuchowicz & Rydzyński, 1998 ▸). As a continuation of our recent work on compounds belonging to the morpholine family (Chidambaranathan *et al.*, 2023 ▸) we are now using morpholine as ligand for coordination complexes. The current study describes the synthesis, crystal structure, Hirshfeld surface, and infrared spectroscopy of bis­[4-(2-amino­eth­yl)morpholine-κ^2^ *N*:*N*′]di­aqua­nickel(II) dichloride.

## 2. Structural commentary

**Figure 1:**
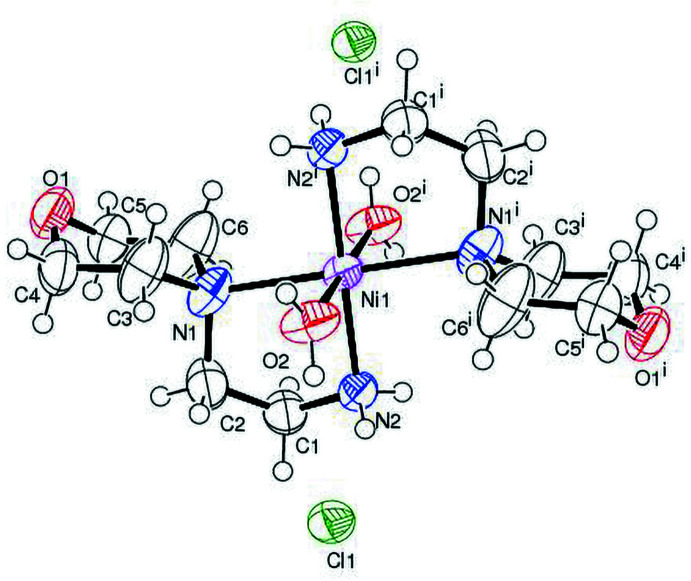
*ORTEP* diagram of the title compound with the atom-numbering scheme. Ellipsoids are drawn at 50% probability. Only the main component of the disordered ligand is shown for clarity. [Symmetry code: (i) −*x* + 1, −*y* + 1, −*z* + 1.]

The title compound (Fig. 1 ▸) crystallizes in the monoclinic space group *P*2_1_/*n* with two complexes in the unit cell. The asymmetric unit comprises one half of an Ni^II^ cation, which is located on an inversion centre, one [(4-(2-amino­eth­yl) morpholine] ligand, one coordinated water mol­ecule and one chloride ion outside the metal coordination sphere. The nickel ion is in an octa­hedral environment of the type N_4_O_2_; the coordination sphere comprises two *N*,*N*′-bidentate morpholine ligands defining the equatorial plane, which form two five-membered chelate rings with the metal centre (Suleiman Gwaram *et al.*, 2011 ▸). The two remaining *trans* axial positions are occupied by the oxygen atoms from the water mol­ecules. As a result of symmetry, the N2—Ni—N2^i^, N1—Ni—N1^i^ and O2–Ni–O2^i^ angles are 180° [symmetry code: (i) −*x* + 1, −*y* + 1, −*z* + 1] and the axes of the octa­hedron are almost perpendicular to each other [N2—Ni—O2 = 90.80 (12), N2—Ni—O2^i^ = 89.20 (12) and O2—Ni—N1^i^ = 91.86 (14)°]. The morpholine rings adopt a chair conformation·The Ni—N (amine) distances, Ni—N1 and Ni—N2, are of 2.249 (4) and 2.067 (3) Å, respectively, in good agreement with the values observed in the literature (Chiumia *et al.* 1999 ▸; Chattopadhyay *et al.* 2005 ▸).

## 3. Supra­molecular features

**Figure 2:**
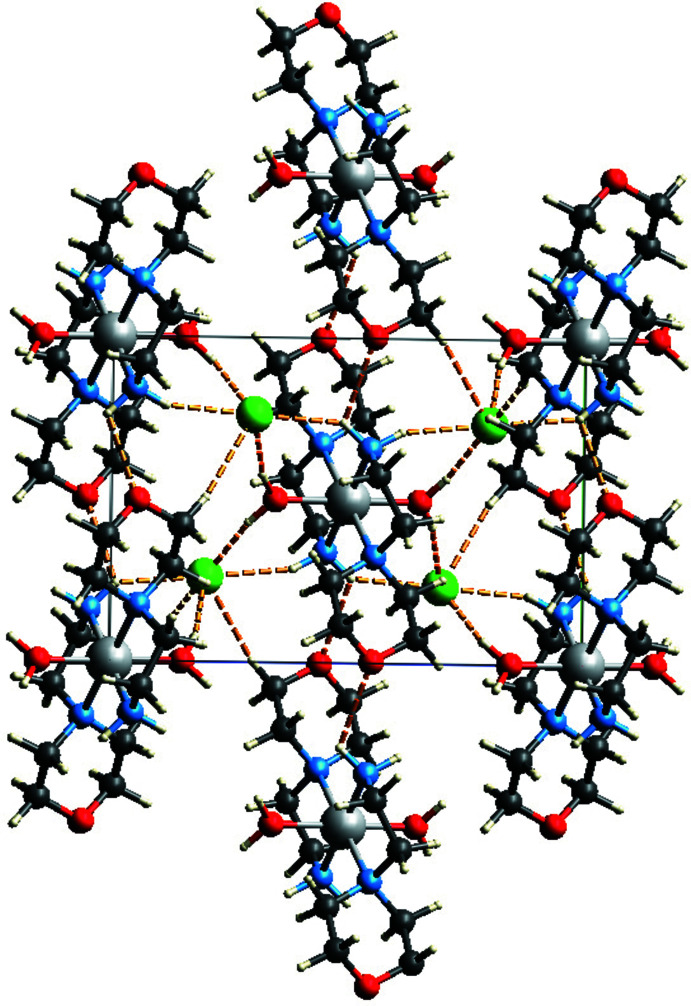
Supra­molecular inter­actions (dotted lines) in the title compound.

Figs. 2 ▸ and 3 ▸ highlight the main supra­molecular inter­actions formed by the title compound (see also Table 1 ▸), while Fig. 4 ▸ shows the overall crystal structure viewed down the *b*-axis direction. In the crystal, the oxygen atoms of the water and of the morpholine mol­ecules (O1 and O2) act as acceptors for several inter­molecular inter­actions of the types N—H⋯O and C—H⋯O, respectively. The uncoordinated chloride anions act as acceptors to C—H and N—H groups of the morpholine mol­ecules and link the adjacent mol­ecules *via* O—H⋯Cl inter­actions involving the water mol­ecules.

**Figure 3:**
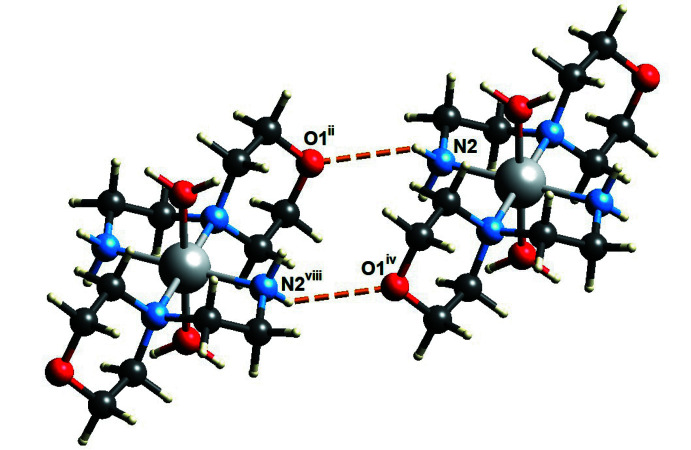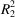 (14) ring motif formed between the two morpholine mol­ecules through very weak N—H⋯O inter­actions (dotted lines). [Symmetry codes ii and iv as in Table 1 ▸. Symmetry code: (viii) −*x* + 1, −*y*, −*z* + 1.]

**Figure 4:**
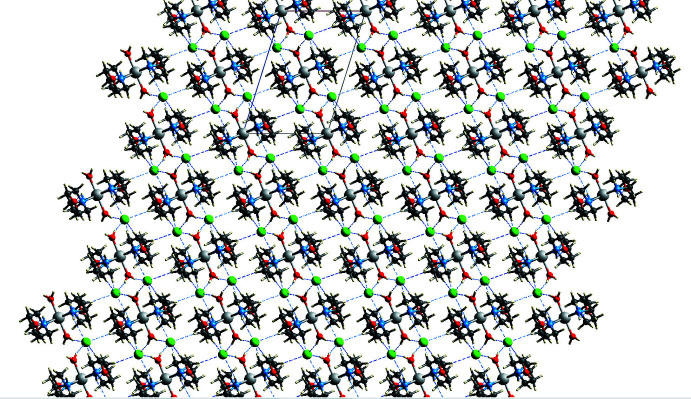
Crystal structure of the title compound viewed down the *b*-axis direction. Dotted lines represent supra­molecular inter­actions.

In particular, a bifurcated inter­molecular hydrogen bond is formed between the N2—H2*E* moiety of the morpholine ligand in the asymmetric unit with an adjacent chloride anion and an adjacent morpholine mol­ecule [N2—H2*E*⋯Cl1^i^ = 2.79 Å and 138.6°; N2—H2*E*⋯O1′^ii^ = 2.38 Å and 126.3°; symmetry codes: (i) *x* − 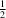 , −*y* + 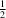 , *z* − 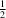 ; (ii) *x*, *y* − 1, *z*]. The two symmetry-related water mol­ecules coordinated by the Ni ion inter­act each with two chloride anions [O2—H1*W*⋯Cl1 = 2.23 (2) Å and 166 (4)°; O2—H2*W*⋯Cl1^vii^ = 2.24 (2) and 166 (5)°; symmetry code: (vii) −*x* + 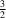 , *y* + 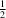 , −*z* + 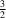 ;] . Finally, graph-set analysis (Spackman *et al.*, 2021 ▸) shows that very weak inter­molecular hydrogen bonds of the type N—H⋯O form an 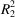 (14) ring motif binding two morpholine mol­ecules into a supra­molecular dimer (Fig. 3 ▸; Bernstein *et al.*, 1995 ▸; Motherwell *et al.*, 2000 ▸; Spackman *et al.*, 2021 ▸).

**Figure 5:**
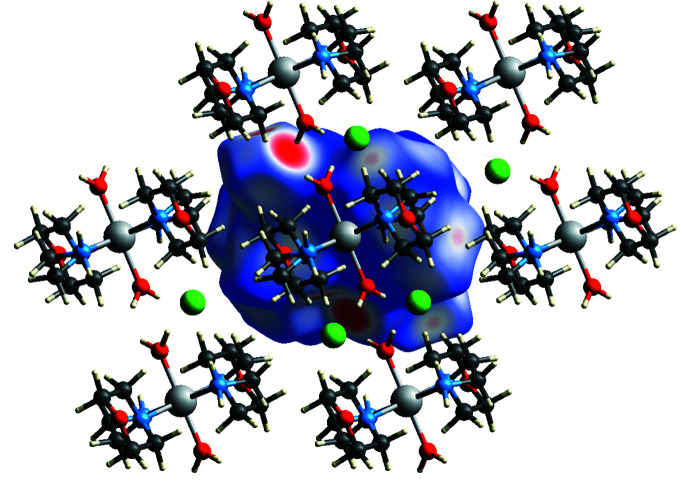
The Hirshfeld surface of the title compound mapped over *d*_norm_, showing the relevant close contacts.

The inter­molecular inter­actions were also studied using Hirshfeld surface analysis, by mapping the normalized contact distances using *CrystalExplorer21.5* (Spackman *et al.*, 2021 ▸). The Hirshfeld surface (HS) was created with a standard surface resolution; the three-dimensional *d*_norm_ surface mapped over a set colour scale ranging from −0.5438 to 1.2671 a.u. is shown in Fig. 5 ▸. In the *d*_norm_ map, blue and red patches show inter­molecular inter­actions with distances greater than and less than the van der Waals radii sum of the inter­acting elements, respectively (Venkatesan *et al.*, 2016 ▸). The brighter red (big circle), lighter red (big circle), lighter red (small circle) and white spots appearing on the HS represent the O—H⋯Cl, N—H⋯Cl, C—H⋯Cl and N—H⋯O inter­actions, respectively.

**Figure 6:**
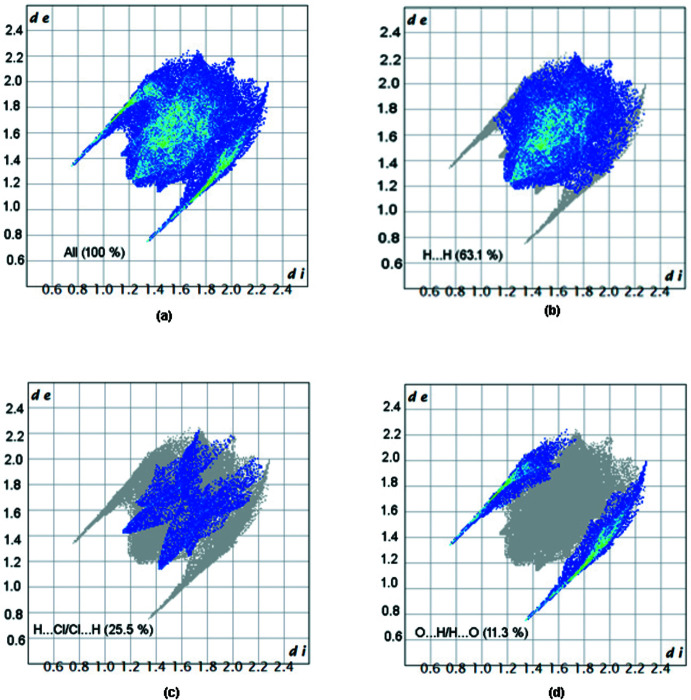
View of the two-dimensional fingerprint plots for the title compound showing (*a*) all inter­actions, and those delineated into (*b*) H⋯H (63.1%), (*c*) H⋯Cl/Cl⋯H (25.5%) and (*d*) O⋯H/H⋯O (11.3%) contacts.

Fig. 6 ▸ shows the two-dimensional fingerprint plots exhibiting (*a*) all inter­molecular inter­actions and those delineated into (*b*) H⋯H, (*c*) H⋯Cl/Cl⋯H and (*d*) O⋯H/H⋯O contacts. The distances from a place on the HS to the closest atoms outside and inside the surface are represented by *d*_e_ and *d*_i_ in the figure. The most important inter­action is H⋯H, which accounts for 63.1% of the total crystal packing and is shown in Fig. 6 ▸ *b* by a pair of symmetrical blunt spikes with points at *d*_e_ + *d*_i_ ∼2.4 Å. The high contribution of these inter­actions suggests that van der Waals inter­actions play a major role in the crystal packing (Hathwar *et al.*, 2015 ▸). The H⋯Cl inter­actions are shown by the presence of a pair of wings in the fingerprint plot shown in Fig. 6 ▸ *c* with the tips at *d*_e_ + *d*_i_ ∼2.5 Å, contributing 25.5% to the HS. The pair of sharp symmetrical spikes in the fingerprint plot delineated into O⋯H/H⋯O contacts (11.3% contribution to the HS, Fig. 6 ▸ *d*), shows a symmetric distribution of points with the tips at *d*_e_ + *d*_i_ ∼2.1 Å.

## 4. Database survey

A search in the Cambridge Structural Database (CSD, version 5.40; Groom *et al.*, 2016 ▸) for the keyword ‘4-(2-amino­eth­yl)morpholine’ yielded nine hits for coordination compounds of 4-(2-amino­eth­yl)morpholine with metals, including *catena*-[bis­(μ_2_-dicyanamide-*N*,*N*′)(4-(2-amino­eth­yl)morpholine]­nick­el(II) (FIJROG; Konar *et al.*, 2005 ▸), bis­[2-(morpholin-4-yl)ethanamine][5,10,15,20-tetra­kis­(4-meth­oxy­phen­yl)porph­yr­inato]iron(II) (NABXEW; Ben Haj Hassen *et al.*, 2016 ▸; NABXEW01; Khélifa *et al.*, 2016 ▸), *trans*-bis­[4-(2-amino­eth­yl)morpholine]­bis­(nitrito)nickel(II) (NAVNAA; Chattopadhyay *et al.*, 2005 ▸; RANVEJ and NAVNAA01; Brayshaw *et al.*, 2012 ▸), *trans*-bis­(iso­thio­cyanato-*N*)bis­[4-(2-amino­eth­yl)morph­oline-*N*,*N*′]nickel(II) (NENSUU; Laskar *et al.*, 2001 ▸), [4-(2-amino­eth­yl)morpholine-*N*,*N*′]aqua­(oxalate-*O*,*O*′)cop­per(II) monohydrate (XAZRUM; Koćwin-Giełzak *et al.*, 2006 ▸) and (μ_2_-oxalato)bis­[4-(2-amino­eth­yl)morpholine]­dicopper(II) diperchlorate (YIKQAK; Mukherjee *et al.*, 2001 ▸). All of the above-mentioned structures are consolidated by hydrogen bonds and contain morpholine rings in a chair conformation.

## 5. Synthesis and crystallization

According to the reaction shown in the scheme, the title compound was synthesized by mixing two moles of 4-(2-amino­eth­yl)morpholine (4.34 g) and one mole of nickel chloride hexa­hydrate (3.96 g) in 100 ml of double-distilled water at 303 K. At room temperature, the solution was allowed to evaporate, yielding plate-like ultramarine blue crystals of the title compound. The FT–IR spectrum of the compound was recorded on a BRUKER FT–IR spectrometer. FT–IR (KBr, cm^−1^): 3455 (*w*, N—H), 2967 (*w*, CH_2_), 1614 (*s*, H_2_O), 1307 (*s*, C—N).

## 6. Refinement

Crystal data, data collection and structure refinement details are summarized in Table 2 ▸. The C-bound H atoms were positioned geometrically (C—H = 0.97 Å) and refined using a riding model with [*U*_iso_(H) = 1.2*U*_eq_(C)]. The water hydrogen atoms, H1*W* and H2*W*, were found in a difference-Fourier map and refined freely.

The morpholine ligand was found disordered over two positions with a site occupancy ratio of 0.708 (8):0.292 (8). The positions of the disordered atoms were identified from difference electron-density peaks and refined using DFIX restraints to achieve the target bond distance of the corresponding atoms. Anisotropic displacement parameters of atoms in the group were restrained to be equal using SIMU restraints with an effective standard deviation of 0.02 Å^2^.

## Supplementary Material

Crystal structure: contains datablock(s) I. DOI: 10.1107/S2056989023001470/xi2027sup1.cif

Structure factors: contains datablock(s) I. DOI: 10.1107/S2056989023001470/xi2027Isup2.hkl

FTIR spectrum. DOI: 10.1107/S2056989023001470/xi2027sup3.pdf

Additional supporting information:  crystallographic information; 3D view; checkCIF report

## Figures and Tables

**Table 1:** Hydrogen-bond geometry (Å, °)

*D*—H⋯*A*	*D*—H	H⋯*A*	*D*⋯*A*	*D*—H⋯*A*
N2—H2*E*⋯Cl1^i^	0.89	2.79	3.509 (3)	139
N2—H2*E*⋯O1^ii^	0.89	2.62	3.138 (6)	118
N2—H2*E*⋯O1′^ii^	0.89	2.38	2.998 (10)	126
N2—H2*F*⋯Cl1	0.89	2.56	3.407 (3)	159
C2—H2*A*⋯Cl1^iii^	0.97	2.94	3.894 (5)	169
C3—H3*D*⋯O2	0.97	2.38	3.120 (7)	133
C6—H6*C*⋯O2^iv^	0.97	2.19	3.002 (8)	140
C4—H4*B*⋯Cl1^v^	0.97	2.84	3.799 (7)	168
C4′—H4′1⋯O1′^vi^	0.97	1.81	2.72 (2)	156
C4′—H4′2⋯Cl1^v^	0.97	2.88	3.827 (16)	167
O2—H1*W*⋯Cl1	0.84 (2)	2.23 (2)	3.054 (3)	166 (4)
O2—H2*W*⋯Cl1^vii^	0.84 (2)	2.24 (2)	3.069 (3)	166 (5)

Symmetry codes: (i) 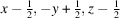 ; (ii) 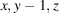 ; (iii) 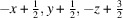 ; (iv) 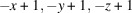 ; (v) 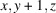 ; (vi) 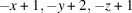 ; (vii) 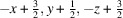 .

**Table 2:** Experimental details

Crystal data	
Chemical formula	[Ni(C_6_H_14_N_2_O)_2_(H_2_O)_2_]Cl_2_
*M*_r_	426.02
Crystal system, space group	Monoclinic, *P*2_1_/*n*
Temperature (K)	298
*a*, *b*, *c* (Å)	8.6495 (4), 8.6593 (4), 13.1882 (6)
β (°)	107.415 (1)
*V* (Å^3^)	942.50 (8)
*Z*	2
Radiation type	Cu *K*α
μ (mm^−1^)	4.30
Crystal size (mm)	0.18 × 0.15 × 0.10

Data collection	
Diffractometer	Bruker D8 VENTURE diffractometer with PHOTON II detector
Absorption correction	Multi-scan (*SADABS*; Bruker, 2016 ▸)
*T*_min_, *T*_max_	0.453, 0.622
No. of measured, independent and observed [*I* > 2σ(*I*)] reflections	15220, 1632, 1564
*R*_int_	0.043
(sin θ/λ)_max_ (Å^−1^)	0.592

Refinement	
*R*[*F* ^2^ > 2σ(*F* ^2^)], *wR*(*F* ^2^), *S*	0.059, 0.165, 1.08
No. of reflections	1632
No. of parameters	143
No. of restraints	113
H-atom treatment	H atoms treated by a mixture of independent and constrained refinement
Δρ_max_, Δρ_min_ (e Å^−3^)	0.70, −0.39

Computer programs: *APEX3*, *SAINT* and *XPREP* (Bruker, 2016 ▸), *SHELXT2018/2* (Sheldrick, 2015*a* ▸), *SHELXL2018/3* (Sheldrick, 2015*b* ▸) and *PLATON* (Spek, 2020 ▸).

## supplementary crystallographic information

### Crystal data

**Table:**

[Ni(C_6_H_14_N_2_O)_2_(H_2_O)_2_]Cl_2_	*F*(000) = 452
*M**_r_* = 426.02	*D*_x_ = 1.501 Mg m^−^^3^
Monoclinic, *P*2_1_/*n*	Cu *K*α radiation, λ = 1.54178 Å
*a* = 8.6495 (4) Å	Cell parameters from 9948 reflections
*b* = 8.6593 (4) Å	θ = 6.2–70.1°
*c* = 13.1882 (6) Å	µ = 4.30 mm^−^^1^
β = 107.415 (1)°	*T* = 298 K
*V* = 942.50 (8) Å^3^	Block, green
*Z* = 2	0.18 × 0.15 × 0.10 mm

### Data collection

**Table:**

Bruker D8 VENTURE diffractometer with PHOTON II detector	1632 independent reflections
Radiation source: micro-focus sealed tube	1564 reflections with *I* > 2σ(*I*)
Graphite monochromator	*R*_int_ = 0.043
ω and φ scan	θ_max_ = 66.0°, θ_min_ = 6.2°
Absorption correction: multi-scan (SADABS; Bruker, 2016)	*h* = −10→10
*T*_min_ = 0.453, *T*_max_ = 0.622	*k* = −10→10
15220 measured reflections	*l* = −15→15

### Refinement

**Table:**

Refinement on *F*^2^	Hydrogen site location: mixed
Least-squares matrix: full	H atoms treated by a mixture of independent and constrained refinement
*R*[*F*^2^ > 2σ(*F*^2^)] = 0.059	*w* = 1/[σ^2^(*F*_o_^2^) + (0.0874*P*)^2^ + 1.7374*P*] where *P* = (*F*_o_^2^ + 2*F*_c_^2^)/3
*wR*(*F*^2^) = 0.165	(Δ/σ)_max_ < 0.001
*S* = 1.08	Δρ_max_ = 0.70 e Å^−^^3^
1632 reflections	Δρ_min_ = −0.38 e Å^−^^3^
143 parameters	Extinction correction: *SHELXL2018/3* (Sheldrick, 2015*b*), Fc^*^=kFc[1+0.001xFc^2^λ^3^/sin(2θ)]^-1/4^
113 restraints	Extinction coefficient: 0.0076 (14)

### Special details

**Table:**

Geometry. All esds (except the esd in the dihedral angle between two l.s. planes) are estimated using the full covariance matrix. The cell esds are taken into account individually in the estimation of esds in distances, angles and torsion angles; correlations between esds in cell parameters are only used when they are defined by crystal symmetry. An approximate (isotropic) treatment of cell esds is used for estimating esds involving l.s. planes.
Refinement. Morpholine moiety is disordered over two positions with a site occupancy ratio of 70:30. The positions of disordered atoms were identified from difference electron density peaks and refined using DFIX restraints to achieve target bond distance of corresponding atoms. Anisotropic displacement parameters of atoms in the group were restrained to be equal using SIMU restraint with an effective standard deviation of 0.02 Å2

### Fractional atomic coordinates and isotropic or equivalent isotropic displacement parameters (Å^2^)

**Table:**

	*x*	*y*	*z*	*U*_iso_*/*U*_eq_	Occ. (<1)
Ni1	0.500000	0.500000	0.500000	0.0377 (4)	
Cl1	0.58426 (12)	0.26230 (11)	0.79999 (7)	0.0497 (4)	
N2	0.3557 (4)	0.3416 (4)	0.5464 (2)	0.0417 (7)	
H2E	0.321086	0.270181	0.496166	0.050*	
H2F	0.412811	0.294617	0.605857	0.050*	
C1	0.2163 (5)	0.4214 (5)	0.5645 (4)	0.0530 (10)	
H1A	0.169116	0.357477	0.607952	0.064*	
H1B	0.134252	0.439523	0.497077	0.064*	
C2	0.2705 (6)	0.5732 (6)	0.6197 (4)	0.0617 (12)	
H2A	0.177632	0.627145	0.629318	0.074*	
H2B	0.346316	0.554100	0.689329	0.074*	
N1	0.3495 (4)	0.6720 (4)	0.5568 (3)	0.0540 (9)	
C3	0.4447 (7)	0.7938 (6)	0.6282 (5)	0.0788 (16)	
H3A	0.503172	0.746613	0.695416	0.095*	0.708 (8)
H3B	0.524266	0.834796	0.596811	0.095*	0.708 (8)
H3C	0.394298	0.808352	0.684009	0.095*	0.292 (8)
H3D	0.551081	0.750267	0.661767	0.095*	0.292 (8)
C6	0.2296 (8)	0.7512 (6)	0.4671 (6)	0.0877 (19)	
H6A	0.285455	0.782072	0.416555	0.105*	0.708 (8)
H6B	0.148727	0.675497	0.431770	0.105*	0.708 (8)
H6C	0.217530	0.688961	0.403994	0.105*	0.292 (8)
H6D	0.126270	0.748890	0.481740	0.105*	0.292 (8)
O1	0.2524 (8)	0.9954 (5)	0.5545 (5)	0.0697 (15)	0.708 (8)
C5	0.1454 (8)	0.8844 (7)	0.4888 (6)	0.0633 (18)	0.708 (8)
H5A	0.086425	0.932949	0.422181	0.076*	0.708 (8)
H5B	0.067047	0.851491	0.523849	0.076*	0.708 (8)
C4	0.3492 (9)	0.9208 (8)	0.6487 (6)	0.0674 (19)	0.708 (8)
H4A	0.278788	0.882257	0.688096	0.081*	0.708 (8)
H4B	0.421466	0.996291	0.693024	0.081*	0.708 (8)
C4'	0.4697 (19)	0.9379 (15)	0.5939 (14)	0.068 (4)	0.292 (8)
H4'1	0.553724	0.933106	0.559085	0.082*	0.292 (8)
H4'2	0.506450	1.006516	0.654532	0.082*	0.292 (8)
C5'	0.254 (2)	0.8918 (14)	0.4431 (12)	0.063 (4)	0.292 (8)
H5'1	0.150028	0.934129	0.402873	0.076*	0.292 (8)
H5'2	0.320186	0.887646	0.395218	0.076*	0.292 (8)
O1'	0.3261 (18)	0.9976 (12)	0.5223 (10)	0.062 (3)	0.292 (8)
O2	0.6639 (4)	0.5015 (4)	0.6529 (3)	0.0588 (9)	
H1W	0.657 (5)	0.441 (4)	0.701 (3)	0.057 (13)*	
H2W	0.738 (6)	0.567 (5)	0.677 (4)	0.10 (2)*	

### Atomic displacement parameters (Å^2^)

**Table:**

	*U*^11^	*U*^22^	*U*^33^	*U*^12^	*U*^13^	*U*^23^
Ni1	0.0404 (6)	0.0349 (6)	0.0345 (5)	−0.0046 (3)	0.0065 (4)	0.0025 (3)
Cl1	0.0538 (6)	0.0504 (6)	0.0434 (6)	0.0047 (4)	0.0124 (4)	0.0080 (4)
N2	0.0451 (16)	0.0373 (16)	0.0409 (16)	−0.0009 (13)	0.0101 (13)	0.0004 (13)
C1	0.048 (2)	0.053 (2)	0.060 (2)	−0.0070 (18)	0.0202 (19)	−0.006 (2)
C2	0.060 (3)	0.060 (3)	0.069 (3)	−0.003 (2)	0.027 (2)	−0.017 (2)
N1	0.0505 (18)	0.0363 (17)	0.066 (2)	−0.0030 (14)	0.0033 (16)	−0.0021 (15)
C3	0.074 (3)	0.049 (3)	0.094 (4)	−0.006 (2)	−0.006 (3)	−0.017 (3)
C6	0.079 (3)	0.054 (3)	0.097 (4)	0.011 (2)	−0.023 (3)	−0.006 (3)
O1	0.080 (4)	0.040 (2)	0.093 (4)	0.002 (2)	0.031 (3)	−0.004 (2)
C5	0.068 (4)	0.049 (3)	0.072 (4)	0.008 (3)	0.020 (3)	−0.001 (3)
C4	0.073 (4)	0.052 (4)	0.080 (4)	−0.007 (3)	0.027 (3)	−0.024 (3)
C4'	0.074 (7)	0.046 (7)	0.079 (8)	−0.009 (6)	0.015 (7)	−0.010 (6)
C5'	0.075 (7)	0.048 (7)	0.065 (7)	0.006 (6)	0.019 (6)	−0.007 (6)
O1'	0.077 (7)	0.041 (5)	0.071 (6)	0.000 (5)	0.024 (5)	0.000 (4)
O2	0.0611 (19)	0.0566 (19)	0.0482 (17)	−0.0190 (14)	0.0003 (15)	0.0115 (13)

### Geometric parameters (Å, º)

**Table:**

Ni1—N2	2.067 (3)	C3—H3D	0.9700
Ni1—N2^i^	2.067 (3)	C6—C5'	1.292 (13)
Ni1—O2^i^	2.090 (3)	C6—C5	1.439 (8)
Ni1—O2	2.090 (3)	C6—H6A	0.9700
Ni1—N1^i^	2.249 (4)	C6—H6B	0.9700
Ni1—N1	2.249 (4)	C6—H6C	0.9700
N2—C1	1.470 (5)	C6—H6D	0.9700
N2—H2E	0.8900	O1—C4	1.428 (9)
N2—H2F	0.8900	O1—C5	1.432 (8)
C1—C2	1.508 (6)	C5—H5A	0.9700
C1—H1A	0.9700	C5—H5B	0.9700
C1—H1B	0.9700	C4—H4A	0.9700
C2—N1	1.492 (6)	C4—H4B	0.9700
C2—H2A	0.9700	C4'—O1'	1.414 (15)
C2—H2B	0.9700	C4'—H4'1	0.9700
N1—C6	1.487 (6)	C4'—H4'2	0.9700
N1—C3	1.487 (6)	C5'—O1'	1.389 (14)
C3—C4'	1.366 (13)	C5'—H5'1	0.9700
C3—C4	1.449 (8)	C5'—H5'2	0.9700
C3—H3A	0.9700	O2—H1W	0.840 (19)
C3—H3B	0.9700	O2—H2W	0.84 (2)
C3—H3C	0.9700		
			
N2—Ni1—N2^i^	180.00 (12)	C4'—C3—H3C	106.5
N2—Ni1—O2^i^	89.20 (12)	N1—C3—H3C	106.5
N2^i^—Ni1—O2^i^	90.80 (12)	C4'—C3—H3D	106.5
N2—Ni1—O2	90.80 (12)	N1—C3—H3D	106.5
N2^i^—Ni1—O2	89.20 (12)	H3C—C3—H3D	106.5
O2^i^—Ni1—O2	180.0	C5'—C6—N1	120.2 (8)
N2—Ni1—N1^i^	96.89 (13)	C5—C6—N1	119.0 (6)
N2^i^—Ni1—N1^i^	83.11 (13)	C5—C6—H6A	107.6
O2^i^—Ni1—N1^i^	88.14 (14)	N1—C6—H6A	107.6
O2—Ni1—N1^i^	91.86 (14)	C5—C6—H6B	107.6
N2—Ni1—N1	83.11 (13)	N1—C6—H6B	107.6
N2^i^—Ni1—N1	96.89 (13)	H6A—C6—H6B	107.0
O2^i^—Ni1—N1	91.86 (14)	C5'—C6—H6C	107.3
O2—Ni1—N1	88.14 (14)	N1—C6—H6C	107.3
N1^i^—Ni1—N1	180.0	C5'—C6—H6D	107.3
C1—N2—Ni1	109.5 (2)	N1—C6—H6D	107.3
C1—N2—H2E	109.8	H6C—C6—H6D	106.9
Ni1—N2—H2E	109.8	C4—O1—C5	109.1 (5)
C1—N2—H2F	109.8	O1—C5—C6	112.6 (5)
Ni1—N2—H2F	109.8	O1—C5—H5A	109.1
H2E—N2—H2F	108.2	C6—C5—H5A	109.1
N2—C1—C2	109.7 (3)	O1—C5—H5B	109.1
N2—C1—H1A	109.7	C6—C5—H5B	109.1
C2—C1—H1A	109.7	H5A—C5—H5B	107.8
N2—C1—H1B	109.7	O1—C4—C3	113.5 (6)
C2—C1—H1B	109.7	O1—C4—H4A	108.9
H1A—C1—H1B	108.2	C3—C4—H4A	108.9
N1—C2—C1	111.1 (4)	O1—C4—H4B	108.9
N1—C2—H2A	109.4	C3—C4—H4B	108.9
C1—C2—H2A	109.4	H4A—C4—H4B	107.7
N1—C2—H2B	109.4	C3—C4'—O1'	111.1 (11)
C1—C2—H2B	109.4	C3—C4'—H4'1	109.4
H2A—C2—H2B	108.0	O1'—C4'—H4'1	109.4
C6—N1—C3	107.4 (4)	C3—C4'—H4'2	109.4
C6—N1—C2	112.3 (4)	O1'—C4'—H4'2	109.4
C3—N1—C2	108.3 (4)	H4'1—C4'—H4'2	108.0
C6—N1—Ni1	112.1 (4)	C6—C5'—O1'	120.4 (13)
C3—N1—Ni1	114.5 (3)	C6—C5'—H5'1	107.2
C2—N1—Ni1	102.3 (2)	O1'—C5'—H5'1	107.2
C4'—C3—N1	123.4 (8)	C6—C5'—H5'2	107.2
C4—C3—N1	114.7 (5)	O1'—C5'—H5'2	107.2
C4—C3—H3A	108.6	H5'1—C5'—H5'2	106.9
N1—C3—H3A	108.6	C5'—O1'—C4'	111.6 (12)
C4—C3—H3B	108.6	Ni1—O2—H1W	123 (3)
N1—C3—H3B	108.6	Ni1—O2—H2W	126 (3)
H3A—C3—H3B	107.6	H1W—O2—H2W	111 (3)
			
Ni1—N2—C1—C2	−40.4 (4)	Ni1—N1—C6—C5'	−104.5 (10)
N2—C1—C2—N1	57.2 (5)	C3—N1—C6—C5	−41.7 (8)
C1—C2—N1—C6	79.2 (5)	C2—N1—C6—C5	77.2 (6)
C1—C2—N1—C3	−162.3 (4)	Ni1—N1—C6—C5	−168.3 (5)
C1—C2—N1—Ni1	−41.1 (4)	C4—O1—C5—C6	−53.8 (8)
C6—N1—C3—C4'	−27.4 (12)	N1—C6—C5—O1	49.0 (9)
C2—N1—C3—C4'	−148.9 (10)	C5—O1—C4—C3	58.6 (8)
Ni1—N1—C3—C4'	97.7 (10)	N1—C3—C4—O1	−56.0 (8)
C6—N1—C3—C4	43.7 (7)	N1—C3—C4'—O1'	42.8 (18)
C2—N1—C3—C4	−77.8 (6)	N1—C6—C5'—O1'	−37 (2)
Ni1—N1—C3—C4	168.8 (5)	C6—C5'—O1'—C4'	50 (2)
C3—N1—C6—C5'	22.1 (12)	C3—C4'—O1'—C5'	−49 (2)
C2—N1—C6—C5'	141.0 (10)		

Symmetry code: (i) −*x*+1, −*y*+1, −*z*+1.

### Hydrogen-bond geometry (Å, º)

**Table:**

*D*—H···*A*	*D*—H	H···*A*	*D*···*A*	*D*—H···*A*
N2—H2*E*···Cl1^ii^	0.89	2.79	3.509 (3)	139
N2—H2*E*···O1^iii^	0.89	2.62	3.138 (6)	118
N2—H2*E*···O1′^iii^	0.89	2.38	2.998 (10)	126
N2—H2*F*···Cl1	0.89	2.56	3.407 (3)	159
C2—H2*A*···Cl1^iv^	0.97	2.94	3.894 (5)	169
C3—H3*D*···O2	0.97	2.38	3.120 (7)	133
C6—H6*C*···O2^i^	0.97	2.19	3.002 (8)	140
C4—H4*B*···Cl1^v^	0.97	2.84	3.799 (7)	168
C4′—H4′1···O1′^vi^	0.97	1.81	2.72 (2)	156
C4′—H4′2···Cl1^v^	0.97	2.88	3.827 (16)	167
O2—H1*W*···Cl1	0.84 (2)	2.23 (2)	3.054 (3)	166 (4)
O2—H2*W*···Cl1^vii^	0.84 (2)	2.24 (2)	3.069 (3)	166 (5)

Symmetry codes: (i) −*x*+1, −*y*+1, −*z*+1; (ii) *x*−1/2, −*y*+1/2, *z*−1/2; (iii) *x*, *y*−1, *z*; (iv) −*x*+1/2, *y*+1/2, −*z*+3/2; (v) *x*, *y*+1, *z*; (vi) −*x*+1, −*y*+2, −*z*+1; (vii) −*x*+3/2, *y*+1/2, −*z*+3/2.
