# Supplementary figures and images for: Synthesis, crystal structure and Hirshfeld surface analysis of bis­[4-(2-amino­eth­yl)morpholine-κ2 N,N′]di­aqua­nickel(II) dichloride
Source: Acta Crystallogr E Crystallogr Commun. 2023 Feb 23;79(Pt 3):226–30. doi: 10.1107/S2056989023001470 (PMC9993921; doi:10.1107/S2056989023001470)

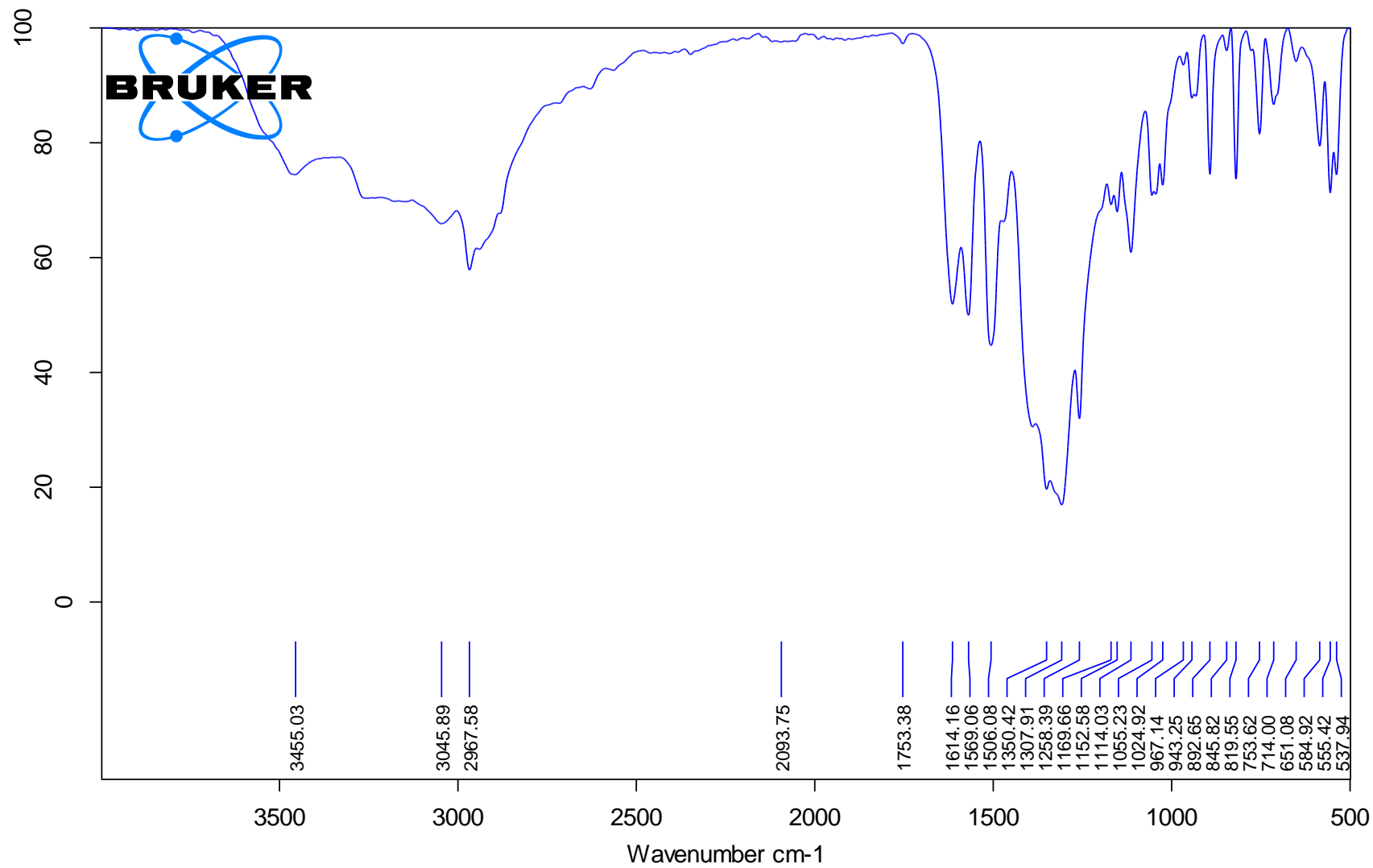

Supplement: Supplementary file 3 [file e-79-00226-sup3.pdf]
